# Catalogues of mammalian long noncoding RNAs: modest conservation and incompleteness

**DOI:** 10.1186/gb-2009-10-11-r124

**Published:** 2009-11-06

**Authors:** Ana C Marques, Chris P Ponting

**Affiliations:** 1 MRC Functional Genomics Unit, University of Oxford, Department of Physiology, Anatomy and Genetics, Oxford OX1 3QX, UK

## Abstract

**Background:**

Despite increasing interest in the noncoding fraction of transcriptomes, the number, species-conservation and functions, if any, of many non-protein-coding transcripts remain to be discovered. Two extensive long intergenic noncoding RNA (ncRNA) transcript catalogues are now available for mouse: over 3,000 macroRNAs identified by cDNA sequencing, and 1,600 long intergenic noncoding RNA (lincRNA) intervals that are predicted from chromatin-state maps. Previously we showed that macroRNAs tend to be more highly conserved than putatively neutral sequence, although only 5% of bases are predicted as constrained. By contrast, over a thousand lincRNAs were reported as being highly conserved. This apparent difference may account for the surprisingly small fraction (11%) of transcripts that are represented in both catalogues. Here we sought to resolve the reported discrepancy between the evolutionary rates for these two sets.

**Results:**

Our analyses reveal lincRNA and macroRNA exon sequences to be subject to the same relatively low degree of sequence constraint. Nonetheless, our observations are consistent with the functionality of a fraction of ncRNA in these sets, with up to a quarter of ncRNA exons having evolved significantly slower than neighboring neutral sequence. The more tissue-specific macroRNAs are enriched in predicted RNA secondary structures and thus may often act in *trans*, whereas the more highly and broadly expressed lincRNAs appear more likely to act in the *cis*-regulation of adjacent transcription factor genes.

**Conclusions:**

Taken together, our results indicate that each of the two ncRNA catalogues unevenly and lightly samples the true, much larger, ncRNA repertoire of the mouse.

## Abstract 2

A comparative evolutionary analysis of two mouse long noncoding RNA libraries reveals a much larger pool of noncoding RNAs remains yet to be discovered.

## Background

The eukaryotic transcriptome now appears far more complex and extensive than previously anticipated. Transcription units are frequently interleaved [1] and transcripts are produced from both coding and noncoding stretches of the genome, including intergenic, intronic and promoter regions [2,3], resulting in a vast array of RNA molecules varying in size, abundance and protein-coding potential. For example, of the 10% of human euchromatic nucleotides that appear to be stably transcribed, more than half lie outside protein-coding gene annotations [2]. Widespread non-protein-coding RNA transcription is evident in many eukaryotic genomes, including mouse, fruitfly and plants [4].

Despite the ever increasing number of long (>200 nucleotide) noncoding RNA (ncRNA) transcripts being identified, the functions of most remain to be determined. Indeed, their biological significance remains controversial [5]. Arguing in favor of their biological relevance are observations that ncRNAs often show variable, perhaps regulated, spatiotemporal expression patterns [6,7], and that their sequences are better conserved with respect to substitutions, insertions and deletions than are putative neutrally evolving sequences [8]. Many long ncRNAs whose functions have been experimentally determined act as transcriptional regulators [4]. For some long ncRNAs it is the act of transcription itself that promotes or suppresses transcription from a neighboring gene locus. In such cases, it is expected that the resultant RNA transcript lacks biological relevance, and thus fails to be subject to selection. The DNA sequence that is transcribed, on the other hand, may be subject to strong selection because of harboring, for example, transcription factor-binding sites that become accessible upon transcription. Other long ncRNAs form ribonucleoprotein complexes that regulate transcription in *trans*, far from the long ncRNA locus. The protein-binding sites of these long ncRNAs, which may span RNA secondary structures, are expected to be subject to purifying selection and thus evolve more slowly than neutral sequence.

In order to address issues of functionality and evolutionary conservation of ncRNAs, it will be important to determine their numbers and conservation (or otherwise) in different eukaryotic genomes. Partial ncRNA catalogues have become available for a number of different species, including mouse. One of the first such catalogues is of long intergenic ncRNA transcripts (herein termed 'macroRNAs') sequenced from mouse cDNA libraries by the FANTOM consortium [3,9]. Comparison of the evolutionary signatures of 3,222 macroRNAs to those of neighboring putatively non-functional sequence showed that macroRNAs exhibit suppressed evolutionary rates for both primary and promoter sequences; these results support the functionality of a small (approximately 5%) portion of such transcripts' sequences [8]. These results countered an earlier analysis that suggested that macroRNAs generally were poorly conserved and thus unlikely to be functional [5]. The findings also serve as an illustration of how the careful application of reliable neutral rates is essential to assess constraint [8].

Recently, Guttman and colleagues [10] introduced a second catalog of 1,600 long intergenic noncoding RNA (lincRNA) intervals in mouse, identified using genome-wide chromatin-state maps. Briefly, lincRNA loci are genomic intervals outside protein-coding gene annotations that are significantly enriched in two epigenetic markers of transcription: trimethylation of lysine 4 on histone H3 (H3K4me3), often associated with active promoters, and trimethylation of lysine 36 on histone H3 (H3K36me3), associated with transcribed regions [11]. In contrast to macroRNA loci, the fraction of transcribed nucleotides and the transcript sequences for most lincRNA intervals remain unidentified. This is because RNA transcription was investigated, using a hybridization-based approach, for only 350 randomly sampled regions [10]. RNA transcription was validated for approximately 70% of these regions, resulting in a total of 2,126 exons overlapping 549 lincRNA intervals [10]. LincRNA exons and their putative promoters were found by Guttman *et al*. [10] to be better conserved than introns, which is suggestive of the action of purifying selection.

In order to compare the level of constraint between macroRNA and lincRNA exons, Guttman and colleagues [12] employed a new method, called SiPhy. Instead of considering the level of constraint across the full extent of these exons' sequences, SiPhy reports exons' highest level of constraint apparent in sliding windows typically of width 12 nucleotides. One advantage of this approach is that it estimates constraint from patterns of nucleotide substitutions. Another is that constraint is estimated across multiple alignments from 21 mammalian species. However, the method does not employ a full evolutionary model. Moreover, comparisons are made against genome-wide random samples, rather than against genomically local samples from putatively neutrally evolving sequence that would account for local substitution rate variation. SiPhy results indicate that lincRNA exons tend to contain more highly constrained 12-nucleotide sequences than macroRNA exons [10]. Rather than calculating maximum constraint, we were interested in assessing the levels of constraint across all aligned exonic nucleotides for each of the two catalogues. We realized that mouse-human pairwise comparisons of full transcript sequences (>100 nucleotides) will often reflect greater numbers of nucleotide substitutions than SiPhy 12-nucleotide windows [12], and thus will tend to be more informative. This analysis would provide a more comprehensive reflection of selective constraints that have acted upon the complete extent of these macroRNA and lincRNA exons.

A surprisingly small fraction (approximately 11%) of lincRNAs were seen to be present also in the macroRNA set. Explanations for this relatively poor overlap were not advanced by Guttman *et al*. [10]. Perhaps the use of different experimental approaches, and of different tissues and cell lines, has resulted in an uneven sampling from different portions of the mouse transcriptome? Alternatively, since the amount of functional sequence and how it compares between catalogues remain unknown, one or both catalogues may contain a large fraction of 'transcriptional noise', resulting from spurious transcription of non-functional loci. If, as Guttman *et al*. report, lincRNA loci are considerably better conserved than are macroRNA loci, then the poor overlap between the two ncRNA catalogues may simply reflect differences in their amounts of inconsequential transcription.

We sought to compare the selective constraints that have acted upon macroRNA and lincRNA loci to determine whether these catalogues contain different proportions of spurious inconsequential ncRNA transcripts. For our analysis we considered the full extent of ncRNA sequences and employed three complementary approaches: a multi-species comparison that complements the Guttman *et al*. approach; a rate-based method that, importantly, accounts for base composition and local variations in neutral substitution rates; and a human population genetics based approach. We considered only exonic sequence - rather than all transcribed ncRNA sequence as done previously [8] - to allow comparison with the Guttman *et al*. study. Contrary to the results of Guttman *et al*., we find that the two ncRNA catalogues exhibit similar degrees of constraint and amounts of functional sequence. We then explored the biological properties of transcripts in these catalogues and concluded that each of these catalogues preferentially samples different portions of the mouse transcriptome. Importantly, our results indicate that a large number of long intergenic ncRNAs remain to be identified.

## Results

### Similar densities of cross-species conserved sequence elements within macroRNA and lincRNA exons

We started by investigating the levels of cross-species conservation for the two long intergenic ncRNA catalogues (macroRNAs [8] and lincRNAs [10]). For this we initially considered whether ncRNA exons are enriched in phastCons elements, short sequences that have been conserved among 17 vertebrate species (including mammals, an amphibian, a bird and fish) [13]. In agreement with the earlier SiPhy results [12], we found that macroRNA and lincRNA exon sets are both significantly overrepresented with such elements (2.2- and 2.6-fold overrepresentations, respectively; *P *< 10^-4^, permutation test; Table 1) compared with intergenic regions. NcRNA exons, from both catalogues, thus contain a higher density of conserved sequence elements than random samples of intergenic sequence, but the two catalogues show no considerable difference in constraint between them.

**Table 1:** Evolutionary signatures and other properties of ncRNAs

	macroRNA	lincRNA
Transcripts or intervals	3,051	1,675
Exons	5,893	2,126
Mouse-human exonic alignments ≥100 bp	3,537	1,784
Median *d*_exon _(mouse-human)	0.418	0.426
Median G+C fraction	0.418	0.430
Median *d*_exon_/*d*_AR _(mouse-human)	0.887	0.904
Fold enrichment of IPSs	1.73	1.77
Fold enrichment of PhastCons 17-way	2.2	2.6
Fold enrichment of Evofold predictions	1.4	0.8
Fold enrichment with transcription factor genes*	1.7	2.4
Rare alleles/intermediate alleles	978/2,675	369/1,110
Constrained exons	1393	595
Promoters	1802	504
Mouse-human promoter alignments ≥100 bp	1477	460
Median *d*_pro _(mouse-human)	0.366	0.402
Median *d*_pro_/*d*_AR _(mouse-human)	0.787	0.857

*Measured as the fold-enrichment within protein coding gene territories (see Materials and methods) associated with the Gene Ontology term 'regulation of transcription'.

These observations were supported by an analysis of the density within ncRNA exons of a second set of conserved sequence, indel-purified sequence (IPS) [14]. This set of 90-Mb long ungapped human, mouse and dog alignments, which is highly enriched in functional sequence, was previously identified using a neutral indel model [14]. By comparing the density of ncRNA exons to the genomic distribution of intergenic IPSs in the mouse genome, macroRNA and lincRNA exons were found to be substantially and significantly overrepresented in IPSs (1.73- and 1.77-fold increases, respectively; *P *< 10^-4^, permutation test; see Materials and methods; Table 1).

### No significant difference in constraint between lincRNA and macroRNA exons

Next we were interested in investigating if macroRNA and lincRNA loci exhibit signatures of constraint on nucleotide substitutions across the full extent of their exonic sequences and whether the lincRNA catalogue, as reported [10], contains highly conserved transcripts. We compared the rates of nucleotide substitution (*d*_exon_) between mouse-human orthologous ncRNA exons to the rates (*d*_AR_) for ancestral repeats (ARs), here defined as transposable elements that inserted prior to the mouse-human split and that remain in both species. Whilst a small minority of transposable elements are clearly functional [15], virtually all (>99.3%) mouse-human ARs have acquired indel mutations uniformly across their sequences, which is the expectation from neutral evolution [14]. Furthermore, highly constrained nonexonic elements arising from transposable elements contribute only 0.04% of the human genome [1]. Consequently, because essentially all ARs evolve neutrally their rate of nucleotide substitution provides a reliable proxy of the neutral rate of evolution [14]. Such neutral rates are strongly autocorrelated on a megabase scale but vary more widely within, and between, chromosomes [16]. Genomically proximal AR sequences thus provide useful evolutionary yardsticks against which the suppression of substitution rates in neighboring functional sequence can be inferred. To ensure the accuracy of estimates, only exons for which at least 100 bp of mouse sequence could be aligned to human were used in this analysis (3,537 of 5,893 macroRNA exons; 1,784 of 2,126 lincRNA exons).

LincRNA exons were observed to exhibit lower substitution rates (median *d*_exon _= 0.426; Table 1) than neighboring neutrally evolving ARs (median *d*_AR _= 0.473). MacroRNA exons also exhibit suppressed rates and to a similar extent (median *d*_exon _= 0.418 versus median *d*_AR _= 0.473; Table 1). These differences in substitution rates between exonic and AR sequences are highly significant (*P *< 10^-15^, two-sided Mann-Whitney (MW) test) and are consistent with a fraction of the ncRNA exons having evolved under selective constraint.

**Figure 1:**
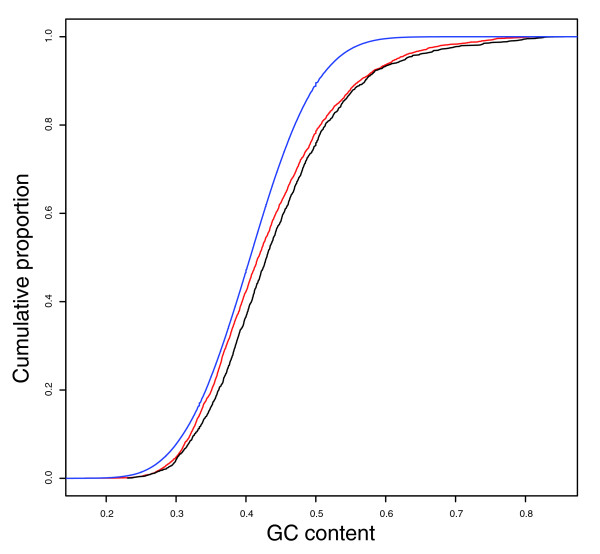
G+C content of mouse ncRNA exons and ancestral repeats. The figure shows the cumulative distribution of G+C fraction as measured for macroRNA exons (red), lincRNA exons (black) and ancestral repeats (blue). LincRNAs tend to have higher G+C contents than macroRNAs. Ancestral repeats tend to possess a low G+C content.

We next sought to compare the extents by which purifying selection has reduced nucleotide substitution rates for the two catalogues, particularly mindful of the claims that lincRNAs are highly conserved, much more so than are macroRNAs [10]. For each catalogue we estimated the degree of constraint on nucleotide substitutions in ncRNA exons versus genomically neighboring ancestral repeats, *d*_exon_/*d*_AR_. The *d*_exon_/*d*_AR _ratio for neutrally evolving sequence is expected not to be significantly different from 1. A ratio significantly smaller than 1 is indicative of purifying selection on deleterious substitutions being more prevalent in ncRNA exons than in closely linked ARs. In this analysis, we took care to account for the known positive correlation of substitution rates with G+C content [17]. This was important because we found that lincRNA exons possess a significantly higher median G+C content than macroRNA exons (43.0% and 41.8%, respectively; *P *< 10^-5^, MW test; Figure 1). We note that this difference in nucleotide composition is likely, through its positive correlation with neutral substitution rate, to contribute to the higher *d*_exon _rate observed for the lincRNA set. It is also notable that the G+C content of ARs neighboring ncRNA loci is significantly lower than that observed for either set of ncRNA exons (median G+C content: 40.6%, *P *< 10^-15^, MW test; Figure 1). To account for these differences, we divided sequences into five distinct classes according to their G+C content (see Materials and methods). For each ncRNA exon the substitution rate (*d*_AR_) of putatively neutral sequence in its vicinity was inferred using mouse-human ARs (within a radius of 200 kb from the ncRNA locus boundary). More specifically, these rates were inferred from AR alignments, matched in length and in nucleotide content to the ncRNA genomic sequence, generated by randomly sampling single columns from ARs that were genomically neighboring and were represented in the same G+C content class.

**Figure 2:**
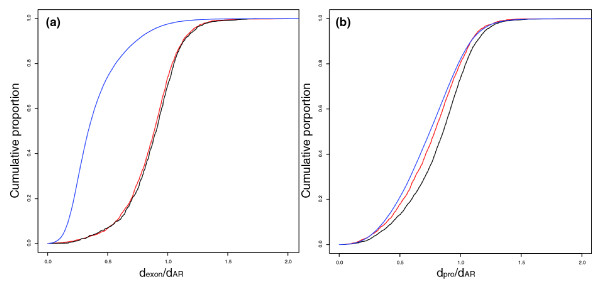
Substitution rates of ncRNA and protein-coding genes. The cumulative distributions of substitution rate for **(a) **exons and **(b) **promoters as measured for macroRNAs (red), lincRNAs (black) and protein-coding genes (blue). MacroRNA and lincRNA exons exhibit similar degrees of constraint and appear to evolve faster than protein-coding exons. Protein-coding gene promoters evolve under stronger constraint than ncRNA exons. MacroRNA promoters have lower substitution rates than lincRNA promoters.

Having accounted for local and G+C-dependent variation in *d*_AR _rates, we observed that median *d*_exon_/*d*_AR _values for lincRNA exons and macroRNA exons were not significantly different (0.904 for lincRNA, and 0.887 for macroRNA; *P *= 0.06, MW test; Table 1 and Figure 2a). In comparison, protein-coding exons were substantially better conserved than these ncRNA sequences (median *d*_exon_/*d*_AR _= 0.338; *P *< 10^-15^, MW test; Figure 2a), in agreement with previous findings [10]. We also compared the numbers of ncRNA exons that individually could be judged as having evolved under constraint owing to a suppressed rate of mouse-human nucleotide substitution rate (see Materials and methods). A significantly higher proportion of macroRNA exons were identified as being constrained compared with lincRNA exons (1,393 (39.4%) versus 595 (33.6%), *P *< 10^-4^, two-sided Fisher's exact (FE) test; Table 1).

We thus conclude, contrary to the initial report [10], first that lincRNA sequences tend not to be highly conserved, and second that their constraint is not significantly different from that of macroRNAs.

### Constraint on orthologous human sequence

We were next interested in whether the human sequences orthologous to the mouse macroRNA and lincRNA exons exhibit a signature of more recent selective constraint, not in the approximately 90 million years since the last common ancestor of human and mouse, but in the last approximately 80,000 years of modern human evolution. For this we took advantage of HapMap data [18] and identified single nucleotide polymorphisms (SNPs) within human sequences orthologous to mouse ncRNA exons and ARs. More specifically, we counted SNPs in macroRNA or lincRNA exons and in ARs according to their derived allele frequencies *f*: SNPs were considered rare if *f *< 10% and intermediate in frequency if 10% ≤ *f *≤ 90%. We then compared proportions of rare derived alleles in ncRNA exons with those in ARs. MacroRNA exons were found to show a significant excess of rare derived alleles, compared to ARs, which is compatible with the action, within the modern human population, of purifying selection on these sequences: numbers of rare over intermediate frequency derived SNPs were 978/2,675 in macroRNAs, and 25,478/79,295 in local ARs (*P *< 0.001, FE test; Table 1). No such biases were found for lincRNA exon sequences (369/1,110 in lincRNAs versus 24,755/77,066 in local ARs, *P *= 0.58, FE test; Table 1). Purifying selection on deleterious mutations during modern human evolution is thus evident for macroRNA exons but not for the lincRNA set.

### Constraint on lincRNA and macroRNA promoter sequences

We then turned to analyzing the evolutionary signatures evident from inferred macroRNA and lincRNA promoter sequences. From the locations of mouse CAGE (cap-analysis of gene expression) tags [3,19] we identified putative transcription start sites (TSSs) overlapping (by at least 1 nucleotide) 504 lincRNA intervals with validated expression and 1,802 macroRNA full-length transcripts. Thereafter we investigated the evolutionary signatures of their associated core promoters, defined as 400 bp upstream of the TSS [20]. To ensure accurate estimates, we only considered putative promoters with an alignable mouse-human region containing greater than 100 bp (460 lincRNA and 1,477 macroRNA promoters).

The median substitution rate within ncRNA promoter (*d*_pro_) regions was found to be significantly lower than that of neutrally evolving local ARs for both ncRNA catalogues (median *d*_pro _values of 0.366 and 0.402 for macroRNAs and lincRNAs, respectively; *P *< 10^-15^, MW test; Table 1). MacroRNA promoters were found to be under significantly greater selective constraint than lincRNA promoters (median *d*_pro_/*d*_AR _values of 0.787 and 0.857, respectively; *P *< 10^-15^, MW test; Table 1 and Figure 2b). Alternatively, these results could reflect a mutational bias resulting in higher mutation rates in lincRNA promoters. In particular, regions of relatively open chromatin (such as active promoters) are prone to higher rates of damage and thus mutation [21]. This mutational effect is expected to be more pronounced for promoters of genes associated with higher levels of expression [21]. As we show later, lincRNAs tend to be expressed at higher levels than macroRNAs.

Both promoter sets appear to have been under slightly lower selective constraints than protein-coding transcript promoters (median *d*_pro_/*d*_AR _= 0.747, *P *< 10^-11^, MW test; Figure 2b). Sequences proximal to TSSs of protein-coding transcripts have previously been shown to be marginally better conserved than those of noncoding transcripts [3].

Our results confirm previous observations [8,10] that ncRNA promoters evolve under stronger purifying selection than their ncRNA transcript sequences (*P *< 10^-7^, MW test). Some of these ncRNAs may thus confer function by virtue of being transcribed, perhaps by altering chromatin status to favor the transcription of genes in their genomic vicinity [22,23], rather than possessing a RNA sequence-specific function.

### Cross-species conservation of transcription

Our findings are consistent with some of these ncRNA loci being functionally conserved across approximately 90 million years of evolution. Nevertheless, for function to be conserved, evidence would be required for transcription in human, as well as mouse, aligned orthologous sequence. It has been reported that approximately 70% of lincRNA intervals in humans exhibit similar chromatin signatures in the orthologous region of the mouse genome [10]. This proportion, however, was only estimated after lincRNA intervals, whose sequences are not alignable between human and mouse, were discarded [10]. This implies that the true fraction of conserved lincRNAs is smaller. Furthermore, as transcription is only expected to be validated for a fraction of these human and mouse lincRNA intervals, it remains to be determined both how many lincRNA loci show conserved transcription in both mouse and human, and whether the two ncRNA catalogues contain different proportions of cross-species conserved (that is, orthologous) ncRNA loci.

To determine the degree of conserved transcription between human and mouse, we searched for evidence of ncRNA exon expression using publicly available expression data (see Materials and methods). The majority of mouse ncRNA loci have no evidence for transcription in their human orthologous sequence, at least from available expressed sequence tag (EST) sequences. Evidence for transcription of human-aligned ncRNA exons was available for only 21% (446) of lincRNAs and 14% (641) of macroRNAs.

Nevertheless, it is notable that lincRNAs exhibit both significantly greater evidence for cross-species conservation of transcription (*P *< 10^-15^, FE test; Table 2) and significantly more exons that can be aligned between human and mouse: 98% of lincRNA exons and 73% of macroRNA exons contain one or more aligned human bases. Interestingly, when compared to the genome of a more closely related species, the rat (*Rattus norvegicus*), similar proportions of (mouse) lincRNA exons (94%) and macroRNA exons (92%) can be aligned. This would be consistent with more macroRNAs than lincRNAs being specific to the rodent lineage.

**Table 2:** Noncoding RNA expression properties

	macroRNA	lincRNA
Conserved exons	4,401	2,103
Conserved transcribed exons	641	446
Exons with expression data [24]	1,111	230
Median AD value	286.4	311.5
Tissue-specific exons	145	15
Median maximum *T*_*S *_value	0.056	0.052

### Noncoding RNA expression profiles

During our identification of ncRNA promoters (described above), we noted that lincRNA TSSs were more frequently supported by CAGE tags than macroRNA TSSs (91% and 59%, respectively, *P *< 10^-16^, FE test). Moreover, lincRNA TSSs are associated with greater numbers of CAGE tags than macroRNA TSSs (means of 6.8 and 5.0 for lincRNAs and macroRNAs, respectively). These two observations suggested that lincRNAs may tend to be expressed at higher levels than macroRNAs. To investigate this hypothesis, we used data from gene expression arrays targeting over 36,000 mouse transcripts [24]. These arrays were found to interrogate 230 lincRNA exons and 1,111 macroRNA exons. We shall assume these subsets of exons faithfully represent the characteristics of the full sets. We compared the median average difference (AD) value between the two catalogues. Between values of 10^2 ^and 10^4 ^AD is known to be proportional to mRNA concentration in the sample [25,26]. We found that lincRNA exons tend to be expressed more highly than macroRNA exons (median AD values of 311.5 and 286.4, respectively; *P *< 5 × 10^-3^, MW test; Table 2). Exons represented in both catalogues, or exons that were found to be constrained, are also more likely to be highly expressed (data not shown).

The higher expression of lincRNAs may indicate that macroRNAs are more likely to be expressed in a tissue-specific manner. To explore this hypothesis, we compared the number of 'tissue-specific' ncRNAs for which the expression level in one tissue was at least five times higher than the median expression level across all tissues. We identified more such tissue-specific ncRNA exons for the macroRNA set compared to the lincRNA set (148 (13%) and 15 (6%), respectively; *P *< 5 × 10^-3^, FE test; Table 2). Similar results were obtained when other tissue-specificity thresholds (namely, three- and ten-fold higher expression level than the median expression level across all tissues) were used (data not shown). Next we calculated tissue specificity (T_*S*_) values for each tissue and each locus. T_*S *_is defined as the fractional expression of a locus in one tissue relative to the sum of its expression in all tissues. The maximum T_*S *_value (maxT_*S*_) for a locus thus provides an indicator of tissue specificity, with higher values reflecting more tissue-specific expression [27]. We found that macroRNA exons do indeed have a higher median maxT_*S *_value than lincRNA exons (0.056 and 0.052, respectively, *P *< 0.01, MW test; Table 2). These findings support the hypothesis that macroRNAs tend to show more restricted tissue expression than lincRNAs.

Protein-coding genes that are widely expressed tend to evolve more slowly than genes with more restrictive tissue expression [28]; nevertheless, this tendency is mild for genes whose products are intracellular [27]. For long ncRNAs, which of course are also intracellular, no positive correlation was observed between median ncRNA expression level and *d*_exon_/*d*_AR _value (rho = -0.099, *P *< 0.05, Spearman correlation).

### Regulation of ncRNA transcription

To further investigate the hypothesis that macroRNAs tend to be more tissue-specific than lincRNAs, we next considered the chromatin status of macroRNA loci. LincRNAs were defined, in part, from their promoters being associated with trimethylation of lysine 4 of histone H3 (H3K4me3) and their transcribed regions being associated with trimethylation of lysine 36 of histone H3 (H3K4me36) [10]. In mouse embryonic stem cells (ESCs) macroRNAs are substantially and significantly (13.9-fold, *P *< 10^-4^, permutation test) enriched in the H3K4me3 epigenetic mark at promoters, but their exons are not associated with the H3K4me36 mark of active transcription (1.2-fold enrichment; *P *= 0.1, permutation test). This suggests that although their promoters are active, macroRNA transcription occurs at low levels in ESCs. This interpretation is consistent with the higher enrichment of H3K27me3, a mark usually associated with transcriptional repression, in the macroRNA promoters than in lincRNA promoters (9.5- versus 4.9-fold enrichment; *P *< 10^-3^, permutation test) for ESCs. Although these data represent only promoter and transcript activity for one cell line, they are consistent with our hybridization-based expression analysis in several mouse tissues. The observed differences in chromatin maps between lincRNA and macroRNA intervals in this one cell type may reflect the higher and broader expression of lincRNAs in many cells and tissues.

### Biological insights onto lincRNA and macroRNA function

The two ncRNA catalogues differ in their extents and tissue specificity of expression. Furthermore, lincRNA exons appear to be more enriched in short highly conserved sequences than macroRNA exons. One contributing reason for the higher density of these conserved sequences within lincRNA loci could be the preference for ncRNA loci to lie in the genomic vicinity of particular classes of protein-coding genes. Transcription factor genes, for example, are typically found in the midst of large numbers of conserved noncoding sequence [29,30]. Both ourselves [31] and Guttman *et al*. [10] have shown a significant tendency for macroRNAs and lincRNAs, respectively, to be transcribed in the vicinity of transcription factor gene loci. This analysis was repeated to allow a direct comparison between these sets. Although we find that macroRNA exons and lincRNA exons each exhibit a significant (*P *< 10^-4^, permutation test) preference to lie close to protein-coding genes involved in the regulation of transcription, this enrichment is considerably stronger for lincRNAs than it is for macroRNA exons (2.4- and 1.7-fold enrichment, respectively; Table 1). This result suggests that constrained sequences involved in the transcriptional regulation of neighboring transcription factor genes may contribute more to levels of constraint in lincRNA exons than to macroRNA exons. In other words, the excess of constraint in lincRNA over macroRNA exons may reflect stronger purifying selection on their DNA, rather than RNA, sequences.

Long ncRNAs, perhaps harboring protein-binding ligands in the form of stable RNA secondary structures, may form ribonucleoprotein complexes that regulate transcription in *trans *[32]. In order to gain further insights into lincRNA and macroRNA mechanisms of action, we assessed the relative abundance of RNA-sequence that is functional in ncRNA transcripts by calculating the densities of predicted RNA secondary structures [33] in lincRNA and macroRNA exons. Whilst a significant (*P *< 0.01, permutation test) 1.4-fold over-representation of these structures was found within macroRNA exons (Table 1), no significant tendency was observed for lincRNA exons (20% depletion, *P *= 0.24, permutation test; Table 1). This result suggests that macroRNAs are more likely to regulate transcription in *trans *by forming ribonucleoprotein complexes, whilst lincRNAs are more often *cis*-regulatory molecules.

### Similar results are obtained for a revised, more conservative ncRNA catalog

The set of macroRNAs was defined originally as FANTOM consortium transcripts lacking protein-coding potential that mapped outside of ENSEMBL protein-coding gene models from an early (May 2004; mm5) mouse genome assembly [8]. We were concerned that improvements to the mouse genome assembly [34] and its gene models (in particular their untranslated region sequences) may necessitate redefinition of some intergenic macroRNAs. Indeed, by discarding all ncRNAs overlapping with current ENSEMBL protein-coding gene annotations (mm9) only 2,214 macroRNAs (covering 4,374 exons) and 1,416 lincRNAs (covering 1,033 exons) remain. In these revised ncRNA sets, only 219 of 2,214 (9.8%) macroRNAs and 159 of 1,416 (11.2%) lincRNAs are represented in both catalogues.

This more conservative definition of ncRNA loci leads, as expected, to slight increases in substitution rates (*d*_exon_, *d*_pro_, *d*_exon_/*d*_AR_, *d*_pro_/*d*_AR_; Table S1 in Additional data file 1), and decreases in expression and tissue-specificity levels (AD, max*T*_*S*_; Table S2 in Additional data file 1). Importantly, however, macroRNA loci and their promoters remain more constrained than lincRNA loci (Table S1 in Additional data file 1). We conclude that both ncRNA catalogues contain an important fraction of functional material and that the observed suppression in evolutionary rates is not principally due to overlap with previously unannotated protein-coding gene exons.

## Discussion

This study, and two that preceded it [8,10], indicate that long intergenic ncRNA loci tend to be less constrained than protein-coding genes, but are more constrained than putatively neutral sequence. Mutations within both promoter and transcribed sequences tend to be deleterious and thus are preferentially purged from the population (Table 1) consistent with a fraction of these ncRNA loci being functional. Evolutionary constraint between mouse and human is evident for 23% of all ncRNA exons that we examined (Table 1). The remaining 77% of exons will be divided, in as-yet undefined proportions, among selectively neutral, or positively selected, sequence and among sequence that is specific to rodents, having been acquired in that lineage or else lost in the primate lineage [35]. Functional long ncRNA loci are likely to arise, and to be lost, at high rates, with substantial divergence rendering sequence similarities between diverse vertebrates indiscernible. This is because of purifying selection being considerably less stringent on these ncRNA loci than it is on protein-coding genes [4].

Our study shows, as expected, that coding exons and their promoters tend to be subject to the greatest degree of purifying selection (*d*_exon_/*d*_AR _= 0.338; *d*_pro_/*d*_AR _= 0.747 (median values)), and again as expected [3,8,10] that promoters of long ncRNAs are better conserved than their exons (*d*_pro_/*d*_AR _= 0.787 versus *d*_exon_/*d*_AR _= 0.887 for macroRNA loci; *d*_pro_/*d*_AR _= 0.857 versus *d*_exon_/*d*_AR _= 0.904 for lincRNA (median values)). However, it was unexpected both that lincRNAs were not 'highly conserved', as previously reported [10], and that macroRNA exons were as well conserved as lincRNA exons (Figure 2). Our results are likely to differ from those of Guttman *et al*. primarily because we have analyzed evolutionary signatures of constraint across the full extent of exonic sequences as opposed to restricting analysis to the most highly conserved short sequence motifs within an exon. The approach used here has similar, or even greater, power to detect constrained ncRNAs as that chosen by Guttman and colleagues. Indeed, for most sequences the information content currently available from 12-nucleotide windows of multiply-aligned sequences (with a maximum branch length of four substitutions per site [12]) is smaller than those for human-mouse pairwise comparisons (0.42 substitutions per site on average) exceeding 100 aligned nucleotides.

Our analyses suggest that lincRNA loci contain a lower, not higher, proportion of functional sequence than macroRNA loci. However, this slight difference may only reflect the contrasting experimental approaches taken when defining each of the two catalogues. By contrast to macroRNA exons, whose boundaries were identified by direct mapping of the sequenced transcript onto the mouse genome and thus are expected to correctly delineate macroRNA exons, lincRNA exons were defined using a hybridization-based approach whose accuracy was strongly dependent on the microarray resolution, resulting in exonic limits that were only defined approximately. A consequence of this experimental imprecision is that the lincRNA exon set will be contaminated by intronic nucleotides, which would be expected to slightly inflate true exonic rate estimates. It would thus not be surprising if true lincRNA exons were to have evolved little differently from macroRNA exons.

**Figure 3:**
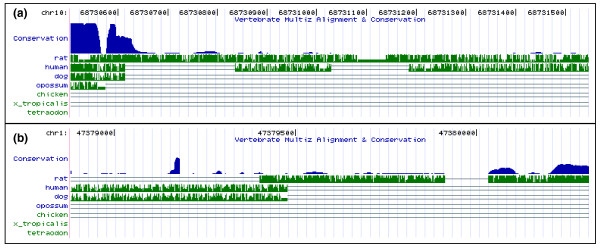
Distribution of highly conserved sequence across ncRNA exon sequences. Examples of phastCons elements (as in [38]) within **(a) **lincRNA (located on chromosome 10, 68730506-68731547) and **(b) **macroRNA (located on chromosome 1, 47378880-47380310) exons. Blue histograms represent the conservation in 17 vertebrates based on a phylogenetic hidden Markov model [13]. Green histograms represent pairwise conservation to other vertebrate species. Images have been taken from the UCSC genome browser.

Guttman *et al*. previously presented evidence that the highest level of constraint in 12-nucleotide windows within lincRNA exons exceeds that within macroRNAs [10]. This would be consistent with the modest difference in multi-species conserved (phastCons) elements between the two sets (Table 1). Nevertheless, a greater proportion of macroRNA exons show significant evidence of constraint than lincRNA exons (Table 1). Taken together, these findings are consistent with lincRNA exons containing short regions of highly constrained sequence, whereas constraint in macroRNA exons is distributed more diffusely (Figure 3). Short functional DNA elements, such as those regulating the expression of transcription factor genes, may contribute more to sequence constraint on lincRNA exons than they have to macroRNA exon constraint. Furthermore, in contrast to macroRNAs, we found no statistical evidence for lincRNAs being enriched in predicted RNA secondary structures. Consequently, macroRNA locus function may more frequently be RNA sequence-specific, whereas lincRNA loci may more often act in a RNA sequence-independent manner, for example, by transcriptional interference [36].

The low degree of concordance between the two sets may reflect a situation where the true long ncRNA locus repertoire of the mouse genome is very large, such that only small numbers of long ncRNA loci are identified in both of two largely independent experimental samples. The low concordance may also reflect biological differences between the two long ncRNA catalogues, which were identified by two very different experimental approaches. MacroRNAs tend to be expressed in a tissue-specific manner and to be associated, in mouse ESCs, with chromatin marks that are usually associated with transcriptional repression (namely, promoter H3K27me3 marks) but not exonic markers of transcription (H3K4me36). The large degree of complementarity between the two catalogues might be expected because macroRNAs are more frequently tissue-specific, having been identified from full-length cDNA libraries prepared from diverse mouse tissues, including from very early embryonic stages and pre-implantation embryos, whereas lincRNA loci were identified from their chromatin marks in four mouse cell types, namely ESCs, embryonic fibroblasts, lung fibroblasts and neural precursor cells. In short, lowly expressed long ncRNAs are more likely to be detected by cDNA sequencing of low abundance transcripts, whereas more highly expressed long ncRNAs are preferentially identified using chromatin status maps.

## Conclusions

Here we show that two sets of intergenic long ncRNA loci in mouse tend to be subject to similar and generally low levels of selective constraint. Nevertheless these sequences evolve significantly slower than neighboring neutral sequence, which is consistent with their functionality.

Importantly, it does appear that the vast majority of all true long ncRNA loci remain to be identified. We arrive at this conclusion because even broadly expressed (that is, non-tissue-specific) macroRNA exons are poorly represented in the lincRNA catalogue (8.4%; 81 of 963 exons). Clearly, much work remains to be done to identify the complete set of mouse ncRNAs before general conclusions concerning their biological mechanisms can be made.

## Materials and methods

### Data sets

We employed the liftOver tool [37] to map successfully onto mouse genome build mm8 3,051 (5,893 exons) of the 3,122 macroRNAs [8] from mouse assembly mm5. We downloaded the genomic positions of lincRNAs and 2,126 lincRNA exons defined by Guttman and colleagues [10]. To identify putative TSSs in both transcript sets we searched for CAGE tags [3,19] (from 592,568 mouse CAGE tag clusters available from the UCSC Genome Browser Database [38]) that overlapped by at least 1 nucleotide the transcribed ncRNA locus. We defined the core promoter as the 400 bp upstream of the putative TSS.

Mouse (mm8) ENSEMBL protein-coding exons were downloaded from the UCSC Genome Browser Database [38]. To define mouse-human ARs, we first downloaded RepeatMasker [39] transposable element annotations from the UCSC Genome Browser Database [38] for human (hg18). Next, human-mouse blastZ whole genome alignments [40], available from the UCSC Genome Browser Database [38], were used to identify and extract the putative orthologous sequence in mouse (mm8) for all loci. All regions of at least 100 bp in size were defined as mouse-human ARs.

### Nucleotide substitution rates

Genomic coordinates of ARs, exons and promoter loci in each catalogue (mm8) were used to obtain their corresponding genomic sequences. Mouse-human blastZ whole genome alignments [40], available from the UCSC Genome Browser Database [38], were used to identify and extract putative orthologous sequence in human (hg18) for all loci. We estimated nucleotide substitution rates (*d*_exon _or *d*_pro_) between orthologous mouse-human aligned sequences using baseml, from the PAML package [41], with the REV substitution model. We considered only mouse-human alignments longer than 100 bp, to ensure the accuracy of the estimates.

The G+C content was determined for all exons, promoters and ARs. AR G+C content was used to define five equally populated classes of G+C content and assigned all sequences to these classes according to their G+C fraction: G+C content ≤ 0.3426; 0.3426 < G+C content ≤ 0.3867; 0.3867 < G+C content ≤ 0.42509; 0.42509 < G+C content ≤ 0.4699; 0.4699 < G+C content. The higher the number of G+C classes the better neutral rates will account for nucleotide content biases but the less neutral material will be available within each class.

To obtain normalized rates (*d*_exon_/*d*_AR _or *d*_pro_/*d*_AR_), we estimated the nucleotide substitution rate for all ARs within 200 kb upstream and downstream of the sequence of interest within the same G+C content class and defined *d*_AR _as the median of these values.

Significantly constrained sequences were identified when their mouse-human substitution rate was significantly (*P *< 0.025, false discovery rate = 3.6%) smaller than the rates of neighboring putatively neutrally evolving sequence with the same nucleotide content. For this, we estimated the local neutral rates by concatenating all local ARs' mouse-human alignments from a matched G+C content class. We then randomly sampled single columns from these alignments to obtain putatively neutral sequence with the exact length and nucleotide content as the sequence of interest and estimated the substitution rate. A promoter or exon was considered to be constrained if fewer than 25 *d*_AR _values from 1,000 iterations of this procedure were smaller than its substitution rate (that is, *P *< 0.025).

### Genome-wide association procedure

The significance of genome-wide associations was assessed, as previously, using a randomization procedure that accounts for G+C content and chromosome-specific biases [8]. This compares, within a workspace *I *(see below), a defined set of genomic segments *S *against multiple randomized sets of segments of matched length *S'*, chosen within the same G+C subset of *I *and within each chromosome. The sets *S *and *S' *are compared with respect to their overlap with six specific sets of sequence annotations *E*: IPSs previously identified with a false discovery rate of 10% [14]; a set of evolutionarily conserved phastCons elements in 17 species (mouse, rat, rabbit, human, chimpanzee, macaque, dog, cow, armadillo, elephant, tenrec, opossum, chicken, *Xenopus*, tetraodon, fugu and zebrafish) alignments (PhastCons17way) [13] obtained from the UCSC Genome Browser Database [38]; Evofold predictions of RNA secondary structure [33]; protein-coding gene territories (see below) associated with Gene Ontology term [42] 'regulation of transcription' [GO:0045449]; H3K4 and H3K27 intervals; and H3K36 intervals. The workspace *I *was defined as the intergenic sequence between ENSEMBL protein-coding genes. In the third case (Evofold predictions of RNA secondary structure) *I *was further restricted to those intergenic sequences that are multiply aligned to genomic sequences of five or more vertebrate species in the eight-way MultiZ alignment [43], and exhibit overlap with PhastCons multispecies conserved elements. This extra filter was required to account for Evofold searching for RNA secondary structures only within such conserved regions. Simulations were performed 10,000 times.

### Protein coding genes with regulation of transcription annotation

We divided the mouse genome into protein-coding gene territories by determining the mid-distance, *i*, between each known mouse protein-coding gene and its closest upstream and downstream protein-coding neighbor *i *- 1 and *i *+ 1 [31]. A gene's territory is defined as the interval delimited by genomic coordinates *i *- 1 to *i *+ 1. Gene ontology annotations for mouse protein-coding genes (build mm8) were downloaded from ENSEMBL [44] and ncRNA loci lying within each territory were associated with this protein-coding gene annotation.

### Derived SNP frequency

We downloaded African population frequency data for SNPs assayed by the HapMap consortium [18], and corresponding chimpanzee and rhesus macaque alleles (NCBI build 1). We considered the ancestral allele to be the chimpanzee allele if its Phred quality score was higher than 40 (less than 1 error per 1,000 bases), or else the macaque allele when it was sequenced at sufficient quality. SNPs were discarded if they failed to segregate within the analysis panel, or if fewer than 100 samples were typed, or if ancestral or reference human alleles (taken from NCBI build 35) failed to agree with either of the SNP alleles. We used whole genome pairwise mouse-human alignments to extract the genomic coordinates of human (hg18) orthologous regions of mouse (mm8) ncRNA exons and ARs. We identified SNPs overlapping these regions and recorded their derived allele frequencies *f*. We compared the numbers of overlapping SNPs within two derived population frequencies, rare (*f *< 10%) or intermediate (10% ≤ *f *≤ 90%), for ncRNA exons and ARs.

### Conservation of expression in orthologous human regions

We used human ESTs and RNA sequences from GenBank to search for evidence of human transcription of orthologous exons of mouse macroRNAs and lincRNAs. The coordinates of all human ESTs and RNAs available from GenBank (downloaded 10 July 2008) that mapped uniquely to regions outside of known protein coding genes in the human genome (Ensembl v50, 384,861 sequences) were mapped onto the mouse build 36 assembly using the human-mouse genome alignment data and the LiftOver [37] tool from UCSC. We used default parameters, and set the minimum ratio of mapped nucleotides to 0.2, appropriate for the human-mouse divergence. We considered a ncRNA exon to be expressed in its human orthologous region if it overlapped by one or more nucleotides one of 145,321 human EST/RNA sequences that map to the mouse genome.

### Expression profile analysis

To investigate the expression profiles of ncRNAs, we used RNA hybridization-based GNF Gene Expression Atlas data for 61 mouse tissues [24]. To associate ncRNA exons with oligonucleotide tags on the microarrays, we intersected their genomic coordinates (obtained from the UCSC Genome Browser Database [38]). Exons were assigned the expression level, measured as AD, of their associated tag. Subsequently, median expression levels for each exon across all 61 tissues were calculated. An exon was classified as being tissue-specific if its expression in one tissue exceeded by at least five times the median expression level across all tissues.

We defined the tissue specificity value (*T*_*S*_) of an exon expressed in tissue *i *as its AD expression value in *i *divided by the sum of its AD values in all tissues. To minimize redundancy in the tissue set, we considered only 32 tissues for which expression had been found previously to be poorly correlated [27]. The maximum *T*_*S *_(max*T*_*S*_) was considered to be the highest *T*_*S *_value found across these 32 tissues.

### Associations with chromatin markers

Genome-wide chromatin-state maps in mouse embryonic stem cells established by chromatin immunoprecipitation followed by sequencing (ChIP-seq) were publicly available [11]. From the Broad Institute webserver [45] we downloaded the genomic intervals, inferred using a hidden Markov model, which are enriched in three chromatin marks: trimethylated histone 3 lysine 4 (H3K4), trimethylated histone 3 lysine 27 (H3K27) or trimethylated histone 3 lysine 36 (H3K36).

### Statistics

Fisher's exact and Mann-Whitney tests were performed using the R package [46,47].

## Abbreviations

AD: average difference; AR: ancestral repeat; CAGE: cap-analysis of gene expression; ESC: embryonic stem cell; EST: expressed sequence tag; FE: Fisher's exact; IPS: indel-purified sequence; lincRNA: long intergenic noncoding RNA; MW: Mann-Whitney; ncRNA: noncoding RNA; SNP: single nucleotide polymorphism; TSS: transcription start site.

## Authors' contributions

ACM and CPP conceived the study. ACM performed all experiments. CPP supervised the study. ACM and CPP drafted the manuscript. All authors read and approved the manuscript.

## Additional data files

The following additional data are available with the online version of this paper: supplementary Tables S1 and S2, which summarize results for macroRNA and lincRNAs with no overlap with current ENSEMBL protein-coding gene annotations (mm9) (Additional data file 1).

## Supplementary Material

Additional data file 1 Results for macroRNA and lincRNAs with no overlap with current ENSEMBL protein-coding gene annotations (mm9). Click here for file
