# Supplementary material for: Catalogues of mammalian long noncoding RNAs: modest conservation and incompleteness
Source: Genome Biol. 2009 Nov 6;10(11):R124. doi: 10.1186/gb-2009-10-11-r124 (PMC3091318; doi:10.1186/gb-2009-10-11-r124)
Supplement: Additional data file 1 — Results for macroRNA and lincRNAs with no overlap with current ENSEMBL protein-coding gene annotations (mm9). [file gb-2009-10-11-r124-S1.DOC]

# Supplementary tables

**Table S1- Summary of ncRNA intergenic in mouse genome (build mm9) evolutionary** signatures

|  | macroRNA | lincRNA |
| --- | --- | --- |
| Transcripts/intervals | 2214 | 1416 |
| Exons | 4374 | 1033 |
| Mouse-human exonic alignments ≥100 bp | 2518 | 864 |
| Median *d*_exon_ | 0.435 | 0.437 |
| Median *d*_exon_/*d*_AR_ | 0.896 | 0.921 |
| Promoters | 1250 | 362 |
| Mouse-human promoter alignments ≥100 bp | 978 | 327 |
| Fold enrichment of Evofold predictions | 1.48 | 0.7 |
| Median *d*_pro_ | 0.410 | 0.433 |
| Median *d*_pro_/*d*_AR_ | 0.858 | 0.904 |

**Table S2- Summary of expression data for ncRNAs whose loci are intergenic in a current mouse genome assembly (build mm9, Ensembl release 43).**

|  | macroRNA | lincRNA |
| --- | --- | --- |
| Exons with expression data [26] | 748 | 89 |
| Median AD | 239.9 | 301.1 |
| Median Maximum *T_S_* value | 0.055 | 0.051 |
